# Distinctive clinical characteristics of malignant mesothelioma in young patients

**DOI:** 10.18632/oncotarget.4414

**Published:** 2015-06-10

**Authors:** Anish Thomas, Yuanbin Chen, Tinghui Yu, Ammara Gill, Vinay Prasad

**Affiliations:** ^1^ Thoracic and GI Oncology Branch, National Cancer Institute, National Institutes of Health, Bethesda, MD, USA; ^2^ Office of Surveillance and Biometrics, Center for Devices and Radiological Health, FDA, Silver Spring, MD, USA; ^3^ Meyer Orthopedic and Rehabilitation Hospital, Springfield, MO, USA; ^4^ Medical Oncology Branch, National Cancer Institute, National Institutes of Health, Bethesda, MD, USA

**Keywords:** mesothelioma, SEER analysis, incidence among the young, asbestos

## Abstract

Although considered a disease of the elderly, a subset of patients with mesothelioma are young (<40 years). The goal of this study was to understand their characteristics and outcomes. The Surveillance, Epidemiology, and End Results (SEER) database was used to extract mesothelioma cases (1990-2010). We modeled Kaplan-Meyer survival curves stratified by site of disease, and age of presentation. 2% (207 of 12345) of mesothelioma patients are young. Sex distribution is comparable among the young (51% males, 49% females); males predominated (78%, 22%) in the older cohort. Frequency of pleural and peritoneal mesothelioma are similar in the young (47%, 48% respectively); pleural disease predominated in the old (90%, 9%). Cancer-directed surgeries are more frequent in the young. Regardless of histologic subtype, young patients with pleural (11 *vs*. 8 months) and peritoneal (not reached *vs*. 10 months) mesothelioma had significantly improved overall survival. In multivariate analysis, younger age was an independent prognostic factor. Although rare, mesothelioma do occur in the young; their characteristics are distinct from those of older patients. Further studies are needed to understand the interplay between genetic susceptibility and mineral fiber carcinogenesis in the pathogenesis of mesothelioma in the young.

## INTRODUCTION

Malignant Mesothelioma is an invasive and often fatal neoplasm that arises from mesothelium that lines several organs. Common primary sites of origin of mesothelioma are the pleura (80–90%) and peritoneum (10–15%) and rarely the pericardium and tunica vaginalis [1]. Among the three main histologic subtypes of mesothelioma, epithelioid tumors are the most common and have a better prognosis than biphasic and sarcomatoid tumors. In patients for whom a macroscopic complete resection is thought to be feasible, mesothelioma is managed with surgery, usually in combination with other modalities of treatment such as chemotherapy or radiation. For patients with unresectable disease, chemotherapy using the regimen of cisplatin plus pemetrexed is the standard of care [2]. The prognosis of patients with unresectable disease is particularly poor with median survival ranging from 10-13 months [2-4].

The majority of cases of mesothelioma are attributed to occupational or environmental asbestos exposure. The link between mesothelioma and exposure to asbestos fibers was elucidated by Wagner et al., in their seminal study of South African miners [5]. The risk of mesothelioma after asbestos exposure continuously increases with time since exposure, and appears to peak 45 years after exposure for pleural mesothelioma, while peritoneal mesothelioma demonstrates no peak, and rises continuously [6]. Asbestos causes DNA damage directly by mechanically interfering with the segregation of chromosomes during mitosis and indirectly by inducing mesothelial cells and macrophages to release mutagenic reactive oxygen and nitrogen species [7]. The incidence of mesothelioma has decreased over several decades in the United States coincident with diminishing occupational asbestos exposure and has remained stable since 2003 [8].

Up to 20% of mesothelioma cases occur in patients without significant exposure to asbestos. Risk factors in this cohort are not well understood, but include radiation exposure [9], exposure to non-asbestos mineral fibers such as erionite [10], simian virus 40 [11], and genetic predisposition [12]. Recent studies have identified germline mutations in the gene encoding BRCA1 associated protein-1 (*BAP1*) which can predispose to mesothelioma [13]. Mesothelioma occurring in germline *BAP1* mutation carriers have been reported to be less aggressive clinically and associated with prolonged survival compared with sporadic mesothelioma [14]. In addition to mesothelioma, germline *BAP1* mutations confer increased susceptibility for the development of several other tumors including uveal melanoma, cutaneous melanoma, renal cell cancers and possibly other cancers [15].

Mesothelioma is often considered a disease of the elderly with median age at presentation of 74 years for pleural and 68 years for peritoneal mesothelioma [16]. Data derived mostly from retrospective studies suggest older age is a poor prognostic factor for mesothelioma [17, 18]. However little is known about mesothelioma in the young. To our knowledge, outside of case studies [19, 20], and small cohorts [21], a detailed examination of this particular subgroup of patients has not been undertaken to date. Considering its long latency period [22], mesothelioma in the young is less likely to be due to occupational exposure to asbestos fibers. These patients may have an increased genetic predisposition to developing mesothelioma or may have environmental exposures to carcinogenic mineral fibers from an early age. Understanding their unique clinical characteristics may provide etiological clues to mesothelioma in this patient population.

The Surveillance, Epidemiology, and End Results (SEER) database provides data from cancer registries from across the United States, serving as a particularly useful tool for studying rare cancers. Utilizing the SEER database, we examined the clinical characteristics and outcomes of young patients with mesothelioma.

## RESULTS

### Patient characteristics

The SEER database included 12345 patients with mesothelioma diagnosed from 1990 to 2010. The younger group (less than 40 years at diagnosis) included 207 (1.7%) patients and the older group (40 and older) 12138 (98.3%) patients.

Table 1 shows the patient characteristics by age group. The sex distribution was comparable among those younger than 40 years (51% males and 49% females), whereas it was male dominant (78% and 22% respectively) among those older than 40 (*p* < 0.0001). In both age groups, mesothelioma was more frequent among whites than other races. Eighty four percent of patients younger than 40 and 92% of older patients were white (*p* = 0.0008). Histological subtype was not available in a majority of cases. Among those with known histology, in both age groups, the most common histological subtype was epithelioid (*p* = 0.012).

**Table 1:** Patient characteristics by age-group

	0-39	>=40	*p* value
	N(%)	N(%)	
Total	207	12,138	
Gender			<0.0001
Men	106(51)	9483(78)	
Women	101(49)	2655(22)	
Race			0.0008
White	173(84)	11121(92)	
Black	20(10)	602(5)	
American Indian/Alaska Native	3(1)	53(1))	
Asian or Pacific Islander	10(5)	342(3)	
Unknown	1(0)	22(0)	
Histology			0.0124
Epithelioid	71(34)	3179(26)	
Sarcomatoid	10(5)	979(8)	
Biphasic	5(2)	625(5)	
Unspecified	121(58)	7355(61)	
Primary Site			
Pleural	98(47)	10863(90)	<0.0001
Peritoneal	99(48)	1127(9)	
Unknown primary site	10(5)	148(1)	

Regarding the primary sites of disease, patients younger than 40 included equal numbers of pleural and peritoneal mesothelioma (47% and 48% respectively), whereas among those older than 40, pleural disease predominated (90% pleural *vs*. 9% peritoneal).

The incidence patterns among patients younger than 40 showed that the frequency of mesothelioma is still a function of age within this group, increasing in older patients (Table 2). Patients between 35 and 39 years accounted for 49% of all young mesothelioma cases. The trend of increasing incidence with increasing age among the young was true for both males and females.

**Table 2:** Age distribution of mesothelioma in patients under 40 years old

	Male	Female	Total
Age at diagnosis	N(%)	N(%)	N
0-4	0(0)	0(0)	0
5-9	1(1)	0(0)	1
10-14	1(1)	1(1)	2
15-19	3(3)	3(3)	6
20-24	2(2)	3(3)	5
25-29	14(14)	19(19)	33
30-34	31(29)	28(28)	59
35-39	54(51)	47(47)	101
Total	106	101	207

From 1990 to 2010, the incidence rate of both males and females under 40 remained low and stable (data not shown). The incidence rate of males above 40 appeared to be slightly decreasing with time when comparing the period of 2001-2010 to the period of 1990-2000. This trend was not statistically significant. The incidence rate of females above 40 remained the same.

### Choice of treatment modalities

In terms of treatments rendered, cancer-directed surgeries were performed more often in the younger patients for pleural mesothelioma: 38% for 0-39 year olds, 32% for 40-64 year olds, and 18% for those older than 65 (Table 3). The difference was statistically significant when comparing those over 65 with the two younger age-groups (*p* < 0.0001). Cancer-directed surgeries were performed more frequently in young patients for peritoneal mesothelioma: 70% for age 0-39 year olds, 47% for 40-64 year olds and 32% for those older than 65 (Table 3). These differences were statistically significant (*p* < 0.0001).

**Table 3:** Cancer-directed surgery performed by age group and stage

	0-39	40-64	65 above	*p* value	*p* value	*p* value
	N(%)	N(%)	N(%)	(0-39 vs. 40-64)	(0-39 vs. >65)	(40-64 vs. >65)
Pleural				0.21	<0.0001	<0.0001
Surgery performed	37(38)	806(32)	1510(18)			
Surgery not performed	56(57)	1633(66)	6679(80)			
Unknown	5(5)	45(2)	190(2)			
						
Peritoneal				0.0001	<0.0001	<0.0001
Surgery performed	69(70)	244(47)	191(32)			
Surgery not performed	30(30)	262(50)	403(67)			
Unknown	0(0)	17(3)	10(2)			

### Clinical outcome

**Figure 1:**
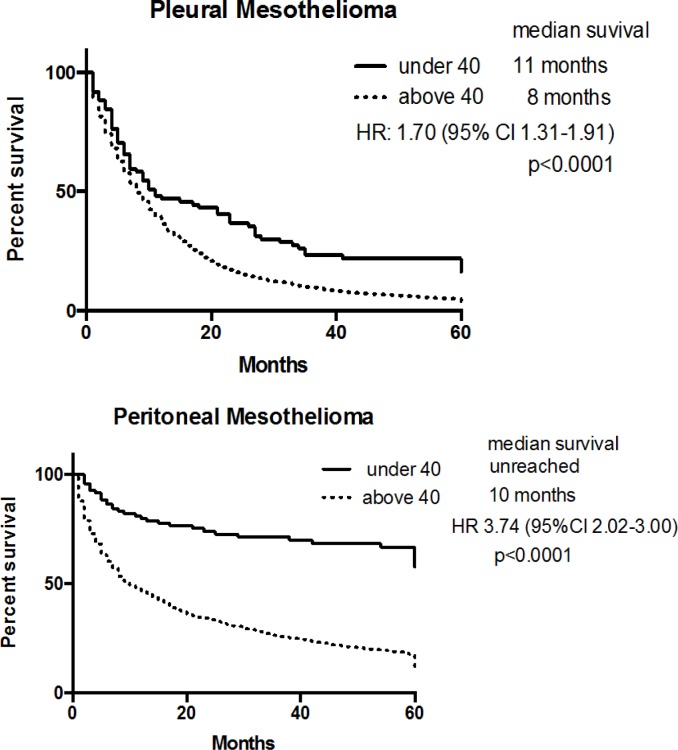
Overall survival of young and old patients by primary site of origin of mesothelioma

Survival analysis was performed using individual patient record for patients who had survival data in the SEER database. Patients under 40 had significantly longer survival compared to the older patients. Regardless of histologic subtype, the median survival time was 34 months *vs*. 8 months, the 5-year survival rate 38% *vs*. 3% in the young and old, respectively. The median survival of the young and old cohorts were 11 months *vs*. 8 months for pleural mesothelioma and was not reached *vs*. 10 months for peritoneal mesothelioma (Figure 1).

### Prognostic factors

To identify potential factors associated with survival, univariate and multivariate analysis were performed using individual records (Table 4). The analysis confirmed that young age was associated with better survival (5 year survival rate 38% *vs*. 3%, 1 year survival rate 65% *vs*. 37%). Hazard ratio for death for patients older than 40 was 3.2 by univariate analysis and 2.5 by multivariate analysis after adjusting for other factors. Female gender, peritoneal histology, receipt of site-directed surgery, and radiation were also associated with better survival. Patients diagnosed between 2001 and 2010 also had better survival than those diagnosed between 1990 and 2000. The hazard ratio for death among patients presenting from 2001 to 2010 compared with 1990 to 2000 was 0.92 and 0.93 in univariate and multivariate analysis, respectively.

**Table 4:** Univariate and multivariate analysis for factors associated with survival

	N	Median survival	1-yr survival rate(95% CI)	5-yr survival rate(95% CI)	Hazard ratio (95% CI)	p value	Hazard ratio (95% CI)	p value
					Univariate analysis	Univariate analysis	Multivariate analysis	Multivariate analysis
**Age group**								
0-39	182	34	64.7 (58.1. 72.2)	38.0 (30.8.46.9)	1		1	
Above 40	9434	8	37.4 (36.4. 38.4)	3.0 (2.6. 3.5)	3.159 (2.592. 3.85)	< 2e-16	2.478 (2.028. 3.029)	<2e-16
								
**Gender**								
Men	7412	8	36.1 (35.0. 37.2)	2.4 (2.0. 2.9)	1.343 (1.275. 1.414)	<2e-16	1.246 (1.183. 1.313)	2.22e-16
Women	2204	9	44.1 (421 46.3)	7.8 (6.6.9.2)	I		1	
								
**Race**								
White	8768	8	37.98 (36.96. 39.03)	3.39 (2.97. 3.88)	1		I	
Black	487	7	36.18 (32.05, 40.84)	5.37 (3.41.8.46)	0.989 (0.896, 1.091)	0.823	1.003 (0.909. 1.106)	0.956
American Indian/Alaska Native	49	8	33.23 (22.17, 49.8)	NA	0.988 (0.732, 1.334)	0.939	1.085 (0.804. 1.465)	0.593
Asian or Pacific Islander	295	8	39.12 (33.81. 45.1)	6.71 (4.19. 10.7)	0.942 (0.831.1.066)	0.343	0.990 (0.874. 1.121)	0.869
Unknown	17	9	35.6 (8.0. 70.6)	NA	0.689(0.391. 1.215)	0.198	0.714 (0.405. 1.257)	0.243
								
**Primary Site**								
Peritoneal	1040	12	50.0 (47.0. 53.2)	15.2 (12.8. 18.0)	I		1	
Pleural	8538	8	36.48 (35.45. 37.53)	2.31 (1.96, 2.73)	1.709 (1.587. 1.841)	< 2e-16	1.525 (1.424. 1.645)	<2e-16
Unknown	38	6	27.7	NA	1.926	0.000227	1.849	0.0005
			(16.33. 46.9)		(1.359. 2.729)		(1.305, 2.620)	
								
**Site-directed surgery**								
Yes	2377	14	55.22 (53.73. 573)	8.84 (7.58. 10.3)	0.5544 (0.5267, 0.5835)	<2e-I6	0.6148 (0.5807. 0.6510)	<2e-16
No	7200	7	32.12 (31.04. 33.24)	1.92 (1.58. 2.34)	I		1	
								
**Radiation**								
Yes	516	16	60.52 (36.36. 65.0)	3.94 (235. 6.6)	0.639 (0.581. 0.704)	<2e-16	0.614 (0.558. 0.677)	<2e-16
No	9100	8	36.62 (35.63. 37.64)	3.65 3.	1		I	
				(3.22. 4.14)				
								
**Year of diagnosis**								
1990-2000	3376	8	36.11 (34.52. 37.77)	2.34 2. (1.88.2.91)	1		1	
2001-2010	6240	8	38.93 (37.71, 40.19)	5.06 (4.40. 5.82)	0.9159 (0.877. 0.957)	0.00008	0.927 (0.887. 0.968)	0.000633

## DISCUSSION

This study provides a comprehensive description of mesothelioma in the young including frequency, time-trends, treatments, outcomes and prognostic factors. We hypothesized that patients diagnosed with mesothelioma when they are younger than 40 years old are a unique subgroup.

Our data shows that approximately 2% of mesothelioma cases in the United States are diagnosed in patients younger than 40. This subgroup indeed has distinctive clinical characteristics. Compared to the older cohort, mesothelioma in the young was associated with lower male to female ratio and comparable frequencies of pleural and peritoneal mesothelioma. Further, they were more likely to undergo cancer-directed surgeries. Regardless of the histologic subtype, young patients with both pleural and peritoneal mesothelioma had significantly improved overall survival compared with older patients. In multivariate analysis, younger age was an independent prognostic factor. Other variables associated with a favorable prognosis included female gender, peritoneal histology, receipt of site-directed surgery, and radiation. The incidence of mesothelioma in the young remained stable between 1990 and 2010.

Mesothelioma of occupational origin has a prolonged latent period with an estimated median latent period of at least 32 years after the initial exposure [22]. Our data, in particular the distribution of sex i.e. comparable incidence in males and females, and primary site of origin of tumor i.e. comparable rates of pleural and peritoneal disease, among young mesothelioma patients as compared to older cohorts indicates that mesothelioma in young patients is less likely to be due to occupational exposure to asbestos fibers. Genetic predisposition and or environmental exposure to carcinogenic mineral fibers from an early age are possible etiological factors in these patients [23].

Genetic predisposition to mesothelioma is supported by a large body of literature which suggests that at least in some individuals there may be a genetic basis for developing mesothelioma, which could lead to mesothelioma by itself or cause susceptibility to asbestos or other mineral fiber carcinogenesis [24]. Familial forms of mesothelioma with autosomal dominant inheritance have been reported in the Cappadocia region of Turkey [25]. Pedigree and mineralogical studies indicated that genetic susceptibility to mineral fiber carcinogenesis plays a critical role in the pathogenesis of mesothelioma in these families [12]. Germline *BAP1* mutations were first described in mesothelioma families with no heavy exposure to carcinogens which are known to cause a high incidence of mesothelioma [13]. Germline *BAP1* mutation carriers have an exceptionally high incidence of malignancies, including mesothelioma and uveal melanoma whereas family members who do not carry these mutations do not develop these malignancies. Germline *BAP1* mutation carriers are thought to be highly susceptible to mesothelioma even at modest levels of asbestos exposure that would be considerably less tumorigenic in the general population [26].

Genetic susceptibility alone cannot explain mesothelioma in the young. It is likely that some patients have an inordinate sensitivity to mineral fiber carcinogenesis. In this study, an increasing incidence of mesothelioma with age was observed within the younger population with patients between 35 and 39 years accounting for nearly half of all young mesothelioma cases. Mesothelioma in young patients with occupational and non-occupational asbestos exposure have been documented in case reports [19, 20], and small population cohorts [21]. Additionally mesothelioma after short latency periods, even less than a decade, have been reported to be associated with occupational exposure to asbestos in both young ([0], and adults [27]. Radiographic abnormalities and asbestosis have also been reported in children of individuals with occupational asbestos exposure [28].

Our results show that young patients with mesothelioma have improved survival compared with older patients. These differences in outcome occurred despite the comparable distribution of patients with known histological subtypes and after adjusting for other variables including cancer-directed surgery. However, in approximately 60% cases in both groups, the histologic subtype which is a major prognostic determinant, was not specified. Disparate distribution of histologic subtypes among the age groups may have confounded the survival estimates.

Our finding of improved survival in younger patients is in line with previous studies. In a retrospective study of over 300 patients with mesothelioma and no prior chemotherapy who enrolled in clinical trials, patients younger than 49 years and good performance status had the best survival [2]. In a cohort of adults that had lived in an asbestos mining town during their childhood, Reid et al observed lower mortality rates among individuals exposed to asbestos when they were less than 15 years old compared to adults with similar exposures who were first exposed when they were more than 15 years old [21]. Improved overall survival of the young mesothelioma patients with both pleural and peritoneal mesothelioma indicates that mesothelioma in the young may possibly have a different natural history and indeed be biologically different from mesothelioma in older patients. Genomic and expression studies of tumor samples from young and old patients with mesothelioma will clarify the biological differences between the two cohorts. To our knowledge no such studies have been conducted to date.

Patients diagnosed between 2001 and 2010 had reduced hazard of death than those diagnosed between 1990 and 2000. This improvement in outcomes may be related to advances in diagnostic imaging, the availability of newer treatment options, such as pemetrexed [2], less toxic treatment regimens and progress in the field of supportive care.

There are several limitations to this study. Due to lack of standardized staging information for mesothelioma in SEER, we were unable to incorporate stage information into this analysis. Further, we were unable to ascertain the extent to which known genetic susceptibility mutations may have influenced the presentation at a younger age. Errors of inclusion, exclusion and misclassification could be present in the SEER database. A high proportion of patients in both groups (58% among young and 62% among old) did not have histologic subtype identified. Finally, we examined a single pre-specified cut-off for age, and not others based on our prior hypothesis that cases younger than 40 are biologically distinct from those occurring at older ages. However, a rigid cut-off likely only approximates any difference, and future research could explore whether a biologically distinct subgroup exists among the younger ages.

In summary, mesothelioma occurs in patients younger than 40 years old, although it is rare. The clinical characteristics of mesothelioma in the young are distinct from those observed from the older patients, including demographic distribution, primary site of origin, cancer-directed surgery rates, and outcomes. The major strength of this study is that it draws upon a large, national database, and provides data which contributes to a better understanding of mesothelioma in the young. Further studies are needed to understand the interplay between genetic susceptibility and mineral fiber carcinogenesis in the pathogenesis of mesothelioma in the young.

## MATERIALS AND METHODS

This was a retrospective, population-based study using cases registered in the SEER database made publicly available through online access. Data were retrieved using the Surveillance Research Program, National Cancer Institute SEER*Stat software (seer.cancer.gov/seerstat) version 8.0.4. Informed consent from the study population was not deemed necessary, as the authors had no access to the identities of the patients.

### SEER database

We examined all cases of mesothelioma from the SEER database. Data set of SEER 18 Register Research Data+ Hurricane Katrina impacted Lousiana Cases were used.

We included all patients meeting the following criteria: Site recode ICD-O-3/WHO 2008 mesothelioma; year of diagnosis 1990-2010. The following mesothelioma histology codes were used in this study: epithelioid 9052, sarcomatoid 9051, biphasic 9053, unspecified 9050. Patients were divided into 0-39 or over 40 age groups, which were pre-specified age groups.

We chose the age cutoff of younger than 40 years at diagnosis to define the “younger” cohort. We hypothesized that mesothelioma in this age group is less likely to be due to occupational exposure to asbestos fibers and that this age threshold will help identify patients with genetic predisposition to mesothelioma or environmental exposure to carcinogenic mineral fibers from an early age.

### Statistical analysis

Individual records were collected for univariate and multivariate survival analyses with regard to various covariates of interest. Homogeneity of the covariate distributions was examined using Chi-square tests. For each sub-group of interest, Kaplan-Meier estimates were generated with individual patient records, such that the five-year survival rates and median survival times can be reported from the right censored records. The hazard ratios between patients of various characteristics were estimated using the semi-parametric Cox model adjusted for age and multiple factors supported by appropriate model selection techniques. Statistical analyses were performed using GraphPad Prism 6.0 and R 2.15.
